# Hydrogen ion dynamics and the Na^+^/H^+^ exchanger in cancer angiogenesis and antiangiogenesis

**DOI:** 10.1038/sj.bjc.6601286

**Published:** 2003-10-14

**Authors:** G Orive, S J Reshkin, S Harguindey, J L Pedraz

**Affiliations:** 1 Faculty of Pharmacy, Department of Pharmacy and Pharmaceutical Technology, University of the Basque Country, C Paseo de la Universidad no. 7, 01006 Vitoria, Spain; 2 Department of General and Environmental Physiology, University of Bari, Via Amendola 165/A, 70126 Bari, Italy; 3 Centro Médico La Salud - C Independencia 13, 01005 Vitoria, Spain

**Keywords:** angiogenesis, Na^+^/H^+^ antiporter, pH regulation, oncogenesis, antiangiogenesis

## Abstract

Tumour angiogenesis and cellular pH regulation, mainly represented by Na^+^/H^+^ antiporter exchange, have been heretofore considered unrelated subfields of cancer research. In this short review, the available experimental evidence relating these areas of modern cancer research is introduced. This perspective also helps to design a new approach that facilitates the opening and development of novel research lines oriented towards a rational incorporation of anticancer drugs into more selective and less toxic therapeutic protocols. The final aim of these efforts is to control cancer progression and dissemination through the control of tumour angiogenesis. Finally, different antiangiogenic drugs that can already be clinically used to this effect are briefly presented.

Increasing attention is being paid to the Na^+^/H^+^ antiporter activity and/or to cell dynamics of the hydrogen ion (H^+^) in different areas of cancer research, at both the basic and clinical levels. These attempts are mainly based upon the key role that both H^+^ ion transport and/or intracellular pH (pH_i_) play in multiple aspects of the biology of tumour cells. It has long been demonstrated that elevations in pH_i_ are directly correlated with the activity of many growth factors and oncogenes, DNA synthesis, cell transformation and proliferation, the metastatic process and multiple drug resistance (MDR) (Harguindey *et al*, 1995). On the contrary, cytosolic hyperacidification is a generalised event in programmed cell death at different stages of the apoptotic process, while systemic acidification has been repeatedly considered to be related in a cause — effect manner to the spontaneous regression of human cancer in both animals and humans (Harguindey *et al*, 1995; Matsuyama *et al*, 2000; Rich *et al*, 2000). The latest research in these areas has shown cause — effect relationships between cellular proton dynamics and Na^+^/H^+^ exchanger at different levels of the neoplastic process, from oncogenesis to multiple drug resistance and from cancer regression to treatment.

In this review, we present the most recent and pertinent data showing how these phenomena are related to the pathogenesis and biology of tumour angiogenesis and antiangiogenesis. In an attempt to integrate these subfields, the genetic and microenvironmental aspects of both stimulatory and inhibitory pathways shared by these areas of cancer research are considered.

## ANGIOGENESIS AND HYDROGEN ION DYNAMICS

Since the seminal work by Folkman (1971), it has been well established that unrestricted growth of malignant tumours requires the induction of new capillary blood vessels. This new vessel formation plays a key role not only in tumour growth but also in invasion and metastasis (Liotta *et al*, 1974). In fact, angiogenesis enhances entry of tumour cells into the circulatory system by providing an increased density of immature and highly permeable blood vessels with little basement membrane and fewer junctional complexes than normal mature vessels (Liotta *et al*, 1974). Moreover, increasing experimental evidence suggests that tumoural angiogenesis is directly related to size, grade, invasive behaviour and clinical outcome of several neoplastic diseases. These range from breast cancer (Weidner *et al*, 1991) to non-small-cell lung cancer (Macchiarini *et al*, 1992), melanoma (Foss *et al*, 1996) and gastric cancer (Maeda *et al*, 1995).

The development of new blood vessels is a complex process involving regulation of gene function and extensive interactions between cells, soluble factors and extracellular matrix components. Interestingly, some of these events also play a significant role in the regulation of the hydrogen ion dynamics of tumour cells, a feature that allows the drawing of close parallelisms between the neovascularisation process and the regulation of intracellular acid–base homeostasis. It is well recognised that pathological elevations of pH_i_ induce many specific biological and functional characteristics of malignant cells such as neoplastic transformation, increases in cell detachment, motility, proliferation, permeability and many others (Harguindey *et al*, 1995; Reshkin *et al*, 2000). Indeed, each of these features on its own is an essential step in the formation of a pathological neovasculature network out of pre-existing normal vessels. Notwithstanding these experimental data, it still remains unclear to which degree and at which points do angiogenesis and H^+^ and Na^+^/H^+^ exchanger-related dynamics show an interaction and if both factors are related in a cause-effect manner.

### Oncogenes and tumour-suppressor genes

The induction and maintenance of a tumour blood vessel are largely attributed to the production of angiogenic factors by malignant cells, a process governed by dominantly acting oncogenes (Rak *et al*, 1995). Switching on of a tumour angiogenic programme is triggered as a result of a shift in the balance of stimulating factors induced by oncogenes and inhibiting factors produced by tumour-suppressor genes. It has been demonstrated that some oncogenes alter cell H^+^ dynamics through an increase in pH_i_, mainly as the result of the activation of membrane-bound Na^+^/H^+^ antiporter (Grunicke *et al*, 1988). For example, there is a close association between ras oncogene expression, modulation of angiogenesis in multiforme glioblastoma and the induction and/or upregulation of vascular endothelial growth factor/vascular permeability factor (VEGF/VPF). Overexpression of VEGF/VPF is also correlated with the action of the H-ras gene product p21 in advanced gastric carcinomas (Kim *et al*, 2000). Similarly, the Ha-ras oncogene directly contributes to tumour development and progression of epidermal tumours through increasing neovascularisation. It has long been recognised that H-ras p21 and Ha-ras, acting via a common pathway upon cell H^+^ dynamics, result in cellular alkalinisation and neoplastic transformation, while the proto-oncogene form of Ha-ras, which does not raise pH_i_, induces only a weak mitogenic response (Doppler *et al*, 1987).

Indeed, a pathological cellular alkalinisation has even been deemed to represent the primary and pivotal factor responsible for neoplastic transformation in different settings, at both the basic and clinical levels (Harguindey *et al*, 1995). It is highly significant that this cellular acid–base shift has been recently considered to be the fundamental and specific derangement in cell transformation and cancer aetiopathogenesis (Reshkin *et al*, 2000). Furthermore, in NIH 3T3 fibroblasts expressing the Ha-ras oncogene, pH_i_ was found to be significantly more alkaline than in identical cells not expressing the oncogene (Grunicke *et al*, 1988). This alkalinisation has been considered to be driven mainly not only by the activation of the Na^+^/H^+^ exchanger, but also by Na^+^, K^+^ and Cl^−^- cotransport systems.

Similarly, loss of function of tumour-suppressor genes leads to the deregulation of cell growth, an event thought to play an outstanding role in the development and progression of a high percentage of human malignancies (Ferreira *et al*, 1999). p53 is a classical tumour-suppressor gene. More than 50% of spontaneous human cancers have either lost or mutated p53 function. This gene is known to have many roles, including cell genome protection, cell cycle arrest, facilitating apoptosis and sensitising tumour cells to chemotherapy. It is thought that p53 performs all these different functions by acting as a molecular stress-responsive device. p53 opposes tumour angiogenesis at various levels; for instance, it inhibits the expression of VEGF and enhances the effect of thrombospondin-1, a powerful inhibitor of angiogenesis (Ferreira *et al*, 1999).

The importance of alterations and/or inactivation of p53 has prompted the scientific community to try to answer some of the questions involving the regulation of this gene, mainly why p53 is prone to so many mutations and how the resulting genetic deregulation can be avoided. Recent work by DiGiammarino *et al*. (2002) seems to have shed significant light in this aspect. These authors studied a group of children in southern Brazil exhibiting an elevated incidence of adrenocortical carcinoma. Out of 36 children with this malignancy, 35 were found to harbour an Arg-to-His mutation within the 337 tetramerisation domain of p53, which appears to be necessary for the gene to function as a regulator of cell cycle control and in the induction of apoptosis. Apparently, the mutant tetramerisation domain is less stable than the wild type, being highly sensitive to pH changes in the physiological range. This pH sensitivity led the authors to suggest that the increased pH_i_ detected in these tumours is the final molecular mechanism responsible for the destabilisation and loss of p53 function and subsequent tumour development.

### Hormonal and pharmacological regulators of angiogenesis

A certain number of positive regulators of angiogenesis have been purified over the last few years (Folkman and Klagsbrun, 1987; O'Really *et al*, 1994, 1997). While these substances influence virtually every aspect of the angiogenic cascade, many of them have overlapping and sometimes opposing functions. The pH_i_ is very sensitive to the response cascade resulting from different factors; in fact, Na^+^/H^+^ antiport-mediated cytosolic alkalinisation is induced by some of these upregulators (Harguindey *et al*, 1995).

Interestingly, many of these angiogenic regulators have been observed to have a direct effect on cell acid–base homeostatic mechanisms, all of them in the same alkaline direction (Table 1

**Table 1:** Effect of angiogenic regulations on cell acid–base homeostatic mechnisms

**Factor**	**Effects on angiogenesis**	**Effects on i.c. hydrogen ion dynamics**
IL-1	Upregulates VEGF/VPF and KDR/flk-1 expression	↑ H^+^-ATPasa and Na^+^/H^+^ APA
IL-8	↑ EC migration and proliferation	↑ i.c. pH and cytoplasmic-free Ca^2+^
EGF	↑ EC DNA synthesis, proliferation and migration	↑ Na^+^/H^+^ APA
PDGF	↑ EC DNA synthesis, proliferation and migration	↑ Na^+^/H^+^ APA
G-CSF	↑ EC DNA synthesis, proliferation and migration	↑ i.c. pH elevation through ↑ Na^+^/H^+^ APA
GM-CSF	↑ activation/differentiation programme related to angiogenesis	↑ Na^+^/H^+^ APA
TNF-*α*	↑ EC migration	↑ Na^+^/H^+^ APA
HGF/SF	↑ tumour survival and proliferation	↑ i.c. pH elevation
TGF-*β*	↑ angiogenesis *in vivo*	↑ Na^+^/H^+^ APA
IGF-I	↑ EC migration, tube formation	↑ Na^+^/H^+^ APA
Angiotensin II	↑ gene expression and secretion of VEGF/VPF	↑ Na^+^/H^+^ APA
PGE_2_	↑ EC proliferation	Induces i.c. alkalosis
Insulin	↑ EC growth and tube formation	↑ Na^+^/H^+^ APA
		

↑=stimulation; EC=endothelial cell; i.c.=intracellular; Na^+^/H^+^ APA=Na^+^/H^+^ antiporter activity.

). Among these, the proinflammatory cytokine IL-1 elevates pH_i_ in T cells through activation of the Na^+^/H^+^ antiporter by a mechanism that may involve protein kinase C (Civitelli *et al*, 1989). IL-1 also has numerous effects on the pathogenesis of tissue injury, upregulating the VEGF-KDR/flk-1 system via activation of tyrosine kinases and increasing transcription in both human proximal tubular cells (El-Awad *et al*, 2000) and human colon cancer cells (Akagi *et al*, 1999). This suggests an important role for IL-1 in the process of angiogenesis both in tumour cells and ischaemic hearts (Maruyama *et al*, 1999).

Hepatocyte growth factor (HGF) is another proangiogenic molecule that also induces a pH elevation in primary cultured hepatocytes by stimulating the Na^+^/H^+^ antiporter through a tyrosine kinase–calcium/calmodulin-dependent pathway. Besides its effect on cellular H^+^ dynamics, HGF is closely associated with angiogenesis through its ability to stimulate endothelial cell chemotaxis by inducing the expression of both VEGF and IL-8 (Dong *et al*, 2001) as well as the expression of the Tie-2 receptor ligand and angiopoietin-2. Finally, angiotensin II is another hormone that upregulates VEGF-KDR/flk-1 expression in retinal microcapillary endothelial cells, neuro-2A cells and bovine retinal pericytes, while at the same time also activating the Na^+^/H^+^ antiporter isoform-1 (NHE-1) (Kusuhara *et al*, 1998).

In spite of the fact that most of the angiogenic factors described in Table 1 increase pH_i_ through stimulation of the Na^+^/H^+^ antiporter, further research is still needed to fully elucidate the exact mechanism by which angiogenic factors modulate the activity of Na^+^/H^+^ antiporters.

### Migration and proliferation of endothelial cells

Endothelial cells (ECs) on the inner surface of blood vessels are normally quiescent, maintaining the integrity of the vessels in the adult. However, when these cells are stimulated by angiogenic mediators, they gain the ability to form neovessels. In this way, the neomorphogenetic creation of vessels influences the tumour microenvironment and *vice versa*, giving rise to rapidly growing vascularised tumours and thereby facilitating tumour spread and the formation of metastatic colonies. The granulocyte- and granulocyte–macrophage-colony-stimulating factor stimulates motility of ECs and leucocytes and this has been demonstrated to be dependent on a functional NHE1 (Denker and Barber, 2002). In fact, among the six family members of the NHE antiporter, the NHE-1 isoform is ubiquitously expressed and plays a key role in pH_i_ regulation and cell-volume homeostasis (Putney *et al*, 2002).

Remodelling of the extracellular matrix (ECM) is a prerequisite for the formation of new vessels. In this vein, angiogenesis shows several functional similarities to the process of tumour cell invasion. These include the specific role of integrin-mediated cell attachment during migration and the requirement of protease-mediated remodelling of ECM. Some of the events that also affect H^+^ dynamics are:

#### Proteases

Degradation of ECM is mediated by a large number of proteases, a process where plasmin and plasminogen activators (PA) play an outstanding role. Urokinase-type plasminogen activator (*μ*PA) is a PA that cleaves plasminogen to plasmin, a glycoprotein with a high proteolytic activity. This process affects EC adhesion and migration. It has been demonstrated that amiloride, an NHE-1 blocker, is a powerful inhibitor of angiogenesis, being able to suppress both *in vitro* and *in vivo* the invasive capacity of human breast cancer cells, at least in part through the inhibition of *μ*PA activity (Evans and Sloan-Stakleff, 2000).

Cathepsins are another family of proteases thought to be important in the process of ECM degradation (Turk *et al*, 2002). While degrading ECM, they release growth factors from the matrix only when the extracellular environment is acidic, prompting many to question their biological significance. Tumours are known to have a more acidic extracellular microenvironment than normal tissues. This is probably created and maintained to a large extent by specific plasma membrane ion transporters, particularly NHE-1. The intracellular microenvironment where the cell is in contact with the ECM reaches very low pH levels thus facilitating the action of the acidic proteases (Webb *et al*, 2001). Interestingly, lowering of extracellular pH or the inhibition of transporters that alkalinise the cell (particularly the NHE-1) has been demonstrated to increase the secretion of cathepsin B in tumour cells (Rozhin *et al*, 1994). This feature suggests a positive feedback as a compensatory mechanism to keep protease activity high in less optimal conditions. We have also found that cathepsin D release was greatly increased by the same experimental protocols (data not shown).

#### Integrins

Endothelial cells attach to their underlying basement membrane through integrin receptors that bind ECM proteins. Cell proliferation and migration rely on the capacity of ECs to move along their basement membrane. It is known that ECs, as well as tumour cells under mitogenic stimulation, employ their *α*_v_*β*_3_ and *α*_v_*β*_5_ receptors to migrate on vitronectin and fibronectin. Furthermore, *α*_v_*β*_3_ can inhibit angiogenesis, suggesting that these receptors are crucial in angiogenesis (Eliceiri and Cheresh, 2001). In addition, adhesion of EC integrins to ECM components has been reported to result in significant elevations of pH_i_ leading some authors to conclude that elevation of intracellular pH is a general property shared by many members of the integrin family. In the same line, it has been demonstrated that integrin activation stimulates cellular adhesion and spreading and that this can be blocked by specific inhibition of the NHE-1.

The trans-activation of growth factor receptors by integrins is an emerging field. Recently, an interesting study showed that cell adhesion, presumably through integrins, activates the human HGF receptor, c-Met, while overexpression of c-Met in hepatocytes resulted in hepatocellular carcinoma in mice (Wang *et al*, 2001). Most significantly, this effect of c-Met is independent of binding of HGF, suggesting that integrin-dependent trans-activation could be responsible for tumourigenesis. Thus, trans-activation of growth factor receptors by integrins may lead to dysregulated cellular growth.

## ANTIANGIOGENEIC MOLECULES AND CELLULAR ACID–BASE HOMEOSTASIS

Antiangiogenesis may be defined as the inhibition of new blood vessel formation and growth. The present day growing interest and excitement surrounding antiangiogenesis as a novel approach to anticancer therapy largely originates from the expectation that anti-angiogenenic agents will eventually be selective in inhibiting the formation of tumour vasculature and/or stimulating its collapse, thus become specifically effective against a wide variety of tumours. Since the first successful clinical treatment along this line, several angiogenic inhibitors are being evaluated in clinical trials for their efficacy as anticancer agents (Kerbel and Folkman, 2002). Significantly, while many proangiogenic molecules stimulate Na^+^/H^+^ antiporter activity and/or increase cell pH, several antiangiogenic agents have been shown to act, directly and/or indirectly, on cellular hydrogen ion dynamics, resulting in a tendency towards cell acidification (Table 2

**Table 2:** Effect of antiangiogenic agents on cellular hydrogen ion dynamics

**Drug**	**Effects on angiogenesis**	**Effects on i.c. hydrogen ion dynamics**
Suramin	↓ angiogenesis and growth	H^+^-ATPasa inhibitor
Squalamine	↓ angiogenesis and growth	↓ Na^+^/H^+^ APA
Warfarin	↓ of prostaglandin synthesis	Acidify the cytoplasm of the cell
Sulindac	Induces apoptosis and ↓ tumour angiogenesis	Interaction with H^+^ of the intermembrane
Genistein	↓ tyrosine kinase, EC proliferation and migration and *μ*PA	↓ Na^+^/H^+^ APA
Captopril	↓ of angiogenesis	↓ Na^+^/H^+^ APA
Amiloride	↓ of *μ*PA activity	↓ NHE-1
Edelfosine	↓ of angiogenesis	↓ Na^+^/H^+^ APA
Natriuretic peptides	↓ EC growth and angiogenesis	↓ of i.c. pH recovery
Staurosporine	↓ of angiogenesis	Induces i.c. acidification

↓=inhibition; EC=endothelial cell; i.c.=intracellular; *μ*PA=urokinase plasminogen activator; Na^+^/H^+^ APA=Na^+^/H^+^ antiporter activity; NHE-1=Na^+^/H^+^ antiporter isoform 1.

).

Suramin is an H^+^-ATPase inhibitor that represents a new type of antitumour agent. This drug appears to interfere with multiple steps and mediators involved in angiogenesis. Suramin has been demonstrated to present antitumour activity in patients with Kaposís sarcoma, non-Hodkińs lymphoma, renal carcinoma, adrenal carcinoma and hormone refractory prostate carcinoma. In fact, patients with prostate carcinoma receiving suramin have been reported to present a survival advantage over other phase II trials drugs in prostate carcinoma (Clark and Chabner, 1995). Although its exact mechanism remains unclear, suramin, as well as other similar drugs, may ultimately work through a pH-related mechanism, as has been suggested in the past.

Squalamine is another new, selective and noncytotoxic inhibitor of new blood vessel formation. This antimicrobial aminosterol is postulated to control new vessel growth by selectively inhibiting the Na^+^/H^+^ antiporter isoform 3, NHE-3. While it has been shown to block hydrogen efflux out of the endothelial cell, thus inhibiting cell alkalinisation and proliferation, some authors believe that disturbances of cellular pH regulation are not the basis for the effects of squalamine (Sills *et al*, 1998). Therefore, its principal mechanism for blocking angiogenesis still needs to be elucidated.

Another significant inhibitor of angiogenesis is the drug amiloride. Amiloride has been advanced to be effective not only in the experimental treatment of neovascularisation in malignant tumours but also in chronic proliferative diabetic retinopathy and in ulcer healing (Sipos and Brem, 2000). As discussed above, amiloride is thought to inhibit angiogenesis by one, or both, of the following mechanisms: by blocking Na^+^/H^+^ antiporter activity and/or by inhibiting the *μ*PA–urokinase plasminogen activator receptor (*μ*PAR) complex.

This group of agents that interact with both angiogenesis and hydrogen ion dynamics represent a new hope for cancer treatment (Table 2). Since it is most likely that no single antiangiogenic drug alone will be effective against all types of tumours, and taking into account the importance of the relationships between tumour microenvironment, tumour development, disease progression and response to therapy, these agents offer additional advantages over other conventional antiangiogenic drugs apart from the likelihood of synergy among them. Whichever might be their final mechanism of action as antiangiogenic agents, these drugs appear to significantly push the tumour milieu towards acidifying tumour cell pH_i_ and, perhaps also, the pH_i_ of the surrounding cells. Finally, occasional clinical cases of malignancies successfully treated with these agents have been reported (Vogt and Frey, 1997; Harguindey *et al*, 2002).

## CONCLUSIONS AND FUTURE PERSPECTIVES

This short review represents an attempt of an in-depth analysis on the relationships between two subfields of oncological research heretofore studied as separate entities: intracellular H^+^ dynamics related to Na^+^/H^+^ antiporter activity and angiogenesis research. The ultimate aim is to both increase the depth of understanding of the oncogenic process as a whole and, at the same time, to develop less toxic as well as more effective and selective approaches to the treatment of malignant diseases. A great deal of both basic and clinical data support the hypothesis of a key and specific role of H^+^ dynamics in the origin, development, spread and maintenance of neoplastic disease. In fact, these H^+^ dynamics not only constitute a key hallmark of cancer progression but also determine the evolution of tumour growth.

As has been reported for other inter-related areas of oncological research, in the setting of tumour vascularisation, a certain number of ‘specific’ proangiogenic molecules and antiangiogenic agents appear to share and function through more encompassing and nonspecific pivotal and/or common final pathways that revolve around the regulation of intracellular H^+^ dynamics (Tables 1 and 2). However, more efforts are necessary in order to unveil the relationships among these fields of oncological research, starting from their genetic basis and microenviromental conditions to the development of more effective cancer approaches to the metastatic process, alone or in combination with standard therapies.
